# A Case of Amoxicillin-Induced Drug Reaction With Eosinophilia and Systemic Symptoms (Dress) Syndrome Associated With Significant Reactive Hypereosinophilia (HE): A Case Report

**DOI:** 10.7759/cureus.41646

**Published:** 2023-07-10

**Authors:** Jaha Oh, Amandeep Singh, Maira Fonseca, Monica Muppidi

**Affiliations:** 1 Department of Internal Medicine, NYC Health and Hospitals/Lincoln, New York City, USA; 2 Department of Dermatology, NYC Health and Hospitals/Lincoln, New York City, USA; 3 Department of Oncology, NYC Health and Hospitals/Lincoln, New York City, USA

**Keywords:** eosinophilia, hypereosinophilic syndrome, hypereosinophilia, amoxicillin, dress syndrome

## Abstract

Drug Reaction with Eosinophilia and Systemic Symptoms (DRESS) syndrome is a severe cutaneous drug reaction characterized by a skin rash, eosinophilia, atypical lymphocytosis, and involvement of multiple organs. The mortality rate of DRESS syndrome is moderate, and prompt diagnosis and treatment are essential. When DRESS syndrome is presented with significant hypereosinophilia (HE), it should be differentiated from other conditions that can cause HE through a comprehensive approach to diagnostic evaluation. Amoxicillin has been well-documented as a potential cause of DRESS syndrome. It is important to note that amoxicillin can trigger DRESS syndrome in patients who already have a known allergy to sulfasalazine, as well as when it is administered with a beta-lactamase inhibitor such as clavulanic acid. Here, we describe a case of amoxicillin alone-induced DRESS syndrome associated with significant reactive HE. A 39-year-old female presented with three days of shortness of breath, fatigue, facial swelling, and a generalized maculopapular skin rash. The patient endorsed taking amoxicillin two to three weeks prior to the presentation. Diagnostic tests revealed HE, significant generalized lymphadenopathy on computed tomography (CT) scans of the neck and abdomen, and bilateral interstitial infiltration on a CT scan of the chest suggestive of eosinophilic infiltration. Based on the European Registry of Severe Cutaneous Adverse Reactions (RegiSCAR) scoring system, the case was categorized as "probable" DRESS syndrome related to amoxicillin. High-dose steroids were initiated as the treatment of choice for suspected DRESS syndrome. Other potential causes of HE were investigated and ruled out. The patient showed significant clinical improvement, with the normalization of absolute eosinophil count (AEC) and complete resolution of lung infiltrates on a repeat CT scan of the chest. The case highlights the importance of conducting a comprehensive diagnostic evaluation to differentiate DRESS syndrome from other causes of HE when significant HE is present. Prompt treatment with high-dose steroids is essential in managing patients with severe symptoms associated with DRESS syndrome. It is crucial to consider amoxicillin as a potential trigger for DRESS syndrome, even when there is no history of sulfasalazine allergy or concurrent administration of a beta-lactamase inhibitor.

## Introduction

Drug Reaction with Eosinophilia and Systemic Symptoms (DRESS) syndrome is a delayed, potentially fatal multiorgan systemic idiosyncratic drug reaction, characterized by a maculopapular rash, fever, lymphadenopathy, and involvement of multiple organs [1-5]. Eosinophilia is defined as an absolute eosinophil count (AEC) of more than 0.5 × 10⁹cells/L while hypereosinophilia (HE) requires an AEC of ≥1.5 × 10⁹cells/L. When HE is associated with eosinophilic end-organ damage confirmed by tissue or bone marrow biopsy, it is categorized as hypereosinophilic syndrome (HES) [6]. Reactive HE is the most common variant and is often associated with conditions that can cause eosinophil expansion such as drug reactions, infections (e.g., helminth infections), inflammatory diseases, and certain lymphoid neoplasms [6]. DRESS presenting as reactive HE should be differentiated from other causes of reactive HE, other variants of HE, and HES if there are significant findings on images or laboratory tests suggestive of organ involvement. A comprehensive approach is needed for the diagnostic evaluation, and it’s important to guide further treatments specific to the underlying etiology [7]. If a patient has any life-threatening symptoms, it should prompt empirical treatment with high doses of steroids [8,9]. Regarding the role of amoxicillin in triggering DRESS syndrome, it has been reported that amoxicillin may contribute to the development of DRESS in patients already showing signs of intolerance to sulfasalazine [4]. However, its role in causing DRESS syndrome in individuals with no previous history is unclear [4]. This case suggests that amoxicillin alone could potentially cause DRESS syndrome without association with other drugs.

This article was previously presented as a meeting abstract at the 2023 NYACP Resident/Fellow and Medical Student Forum on May 12, 2023.

## Case presentation

A 39-year-old woman with a past medical history of migraine presented with three days of malaise, fever, shortness of breath, chest pain, and generalized maculopapular morbilliform skin lesions. Initial vital signs revealed a heart rate of 115/minute and a respiratory rate of 30/minute while the body temperature and oxygen saturation were within normal limits. The physical exam findings of bilateral crackles on lung auscultation and regular tachycardia further suggested possible underlying respiratory and cardiovascular involvement. The presence of diffuse maculopapular skin lesions on the extremities, back, and trunk was also significant. Initial laboratory studies (Table 1) were significant for a white cell count of 16.52K/uL with an absolute eosinophil count (AEC) of 6500 cells/mL. Other notable laboratory findings include an elevated troponin level of 0.030 ng/mL, indicating cardiac injury, an elevated D-dimer level of 1440 ng/mL, and elevated markers of inflammation such as an elevated erythrocyte sedimentation rate (ESR) of 63 mm/hr and C-reactive protein (CRP) of 60 mg/L.

**Table 1:** Laboratory results on admission Remarkable for high absolute eosinophil count (AEC) of 6500 cells/mL

	Reference Value	Reference Units	Patient’s Value
White cell count (WBC)	4.8 – 10.8	x 10^3^/mcL	16.42
Hemoglobin	14.0 – 18.0	g/dL	13.1
Hematocrit	42 - 52	%	40.4
Mean corpuscular volume (MCV)	80 - 99	fL	84.7
Platelet (PLT)	150 - 450	x 10^3^/mcL	322
Neutrophil %	44 - 70	%	50.7
Lymphocyte %	20 – 45	%	6.8
Monocyte %	2 - 10	%	1.6
Eosinophil %	1 - 4	%	40%
Eosinophil absolute count	0.1 - 0.4	x 10^3^/mcL	6.57
Basophil%	0.2 - 1.8	%	0.3
Sodium	136 - 145	Mmol/L	134
Potassium	3.5 - 5.1	Mmol/L	4.3
Chloride	98 - 107	Mmol/L	102
Carbon dioxide	22 - 29	Mmol/L	19
Anion gap	8 - 16	Mmol/L	13
Blood urea nitrogen (BUN)	6 - 23	Mg/dL	9.0
Creatinine	0.5 – 0.9	Mg/dL	0.67
Glucose	74 - 109	Mg/dL	66
Calcium	8.4 – 10.5	Mg/dL	8.5
Protein	6.4 - 8.3	g/dL	7.8
Albumin	3.5 - 5.2	g/dL	3.3
Alkaline phosphatase (ALP)	40 -130	U/L	195
Alanine transaminase (ALT)	≤ 41	U/L	45
Aspartate transaminase (AST)	≤ 40	U/L	44
Pro B-natriuretic peptide (Pro-BNP)	≤ 124	pg/mL	770.2
Troponin T	≤ 0.01	ng/mL	0.030
Lactate dehydrogenase (LDH)	135 - 214	U/L	835
C-reactive protein (CRP)	< 0.4	mg/L	60.4
ESR (erythrocyte sedimentation rate)	0 - 15	mm/hr	63
D-dimer	< 230	ng/mL	1,440
Lactic acid	0.5 - 2.2	Mmol/L	1.5
Creatine kinase	20 - 200	U/L	180
Thyroid-stimulating hormone (TSH)	0.27 - 4.20	uIU/mL	0.67

**Figure 1:**
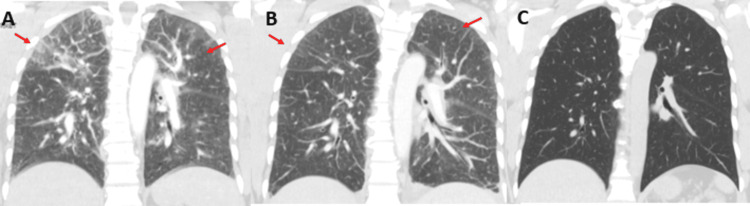
Changes in lung infiltration on CT scan 1A: CT pulmonary angiography. Diffuse bilateral ground-glass and interstitial opacities. Admission day. 1B: CT chest with contrast. Interval decrease in bilateral patchy ground-glass opacities. Admission day 4. 1C: CT chest without contrast. Interval resolution of patchy ground-glass opacities. Twelve days after discharge.

Based on these findings, a computed tomography (CT) lung angiogram was performed to rule out pulmonary embolism, which was negative but remarkable for trace pericardial effusion and diffuse bilateral ground glass interstitial opacities indicating lung involvement. (Figure 1A). The electrocardiogram (ECG) showed sinus tachycardia without ischemic changes. The echocardiogram revealed a normal ejection fraction but an elevated right ventricular systolic pressure, which indicated right ventricular strain, possibly due to lung involvement.@@

**Figure 2:**
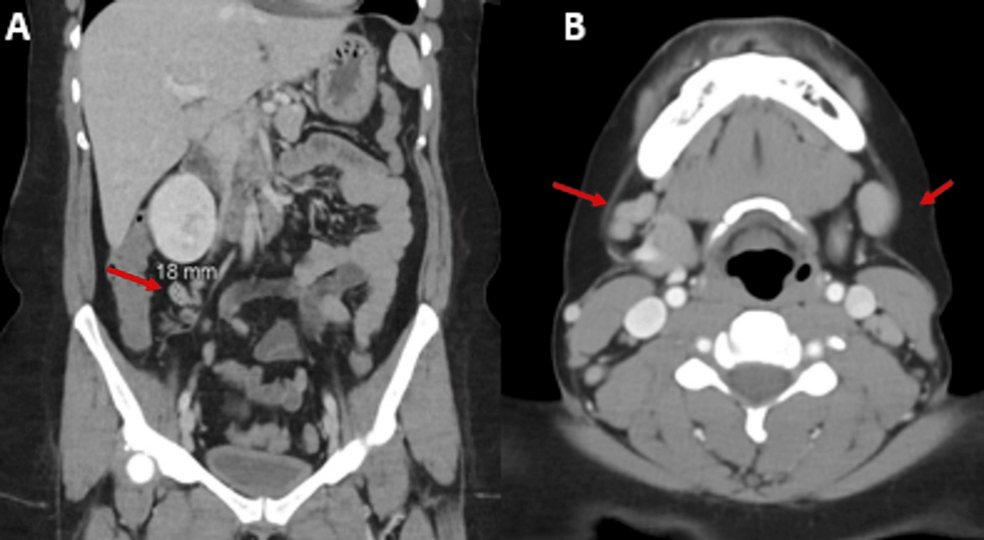
Lymphadenopathy 2A: CT abdomen pelvis with contrast. Increased number of up to 18 mm mesenteric, retroperitoneal, and bilateral lymph nodes. Admission day 4. 2B: CT soft tissue neck with contrast. Bilateral level II and III cervical lymphadenopathies. Bilateral submandibular (level 1B) adenopathy. Left level V adenopathy. Right supraclavicular adenopathy. Admission day 6.

The patient was initially admitted for possible multifocal bacterial pneumonia. Ceftriaxone and azithromycin were started empirically then switched to vancomycin and piperacillin-tazobactam on admission day 4 since the patient was clinically deteriorating. CT chest and CT abdomen and pelvis were performed for further evaluation. Slight interval resolution in lung infiltrations was seen on the follow-up CT chest (Figure 1B). The significant intra-abdominal lymphadenopathies observed on the CT abdomen require further investigation to determine their cause. While no splenomegaly or occult neoplasms were noted, the presence of lymphadenopathies raises concerns about potential underlying conditions such as lymphoproliferative disorders (Figure 2A).

**Figure 3:**
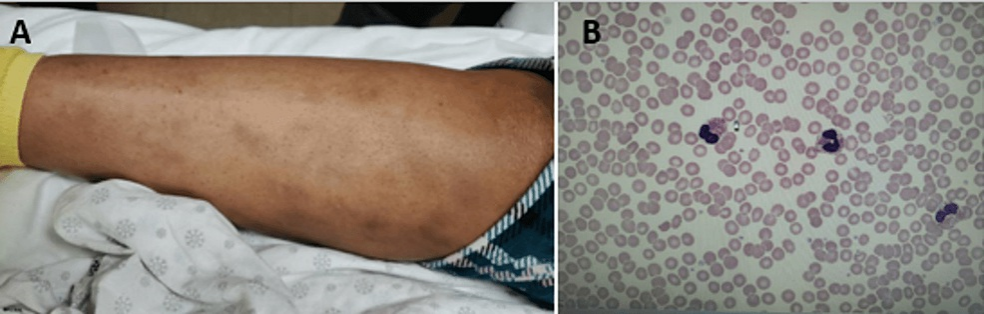
Rash and hypereosinophilia on admission day 6 3A. Pink erythematous macules coalescing into patches on the right leg. Admission Day 6. 3B. Peripheral blood smear. Hypereosinophilia with AEC of 12840 cells/mL. No atypical lymphocytes. Admission Day 6.

Despite antibiotics, the patient’s WBC trended up to 28K/uL with AEC of 15,000 cells/mL on admission day 6, and she complained of neck pain and itchy skin. A physical exam showed noticeable tender cervical lymphadenopathy and faint pink erythematous scattered macules coalescing into patches mainly on the right lower leg (Figure 3A). Peripheral smear showed hypereosinophilia without atypical lymphocytes or blast (Figure 3B). Bilateral level II and III cervical adenopathies were noted on CT soft tissue neck (Figure 2B). On further history taking, the patient reported she took amoxicillin for dental caries two to three weeks prior to this admission, denied prior penicillin or sulfasalazine allergy history but has a known allergy to quinidine with reactions of swelling and rash. Based on this, the patient was suspected to have DRESS syndrome leading to the discontinuation of all antibiotics and a dermatology consult. The RegiScar score (Table 2), which is one of DRESS syndrome's diagnostic criteria, is calculated based on specific clinical features [3]. With a score of 3 for lymphadenopathy, 1 for organ involvement, 2 for eosinophilia, and - 1 for not having a typical rash suggestive of DRESS), the patient falls into the category of 'possible' DRESS syndrome.

**Table 2:** RegiSCAR scoring system for classification of DRESS syndrome Source: [1] Total score: <2: Excluded, 2 to 3: Possible, 4 to 5: Probable, ≥6: Definite DRESS: Drug Reaction With Eosinophilia and Systemic Symptoms; HAV: hepatitis A virus; HBV: hepatitis B virus; HCV: hepatitis C virus; ANA: antinuclear antibody

Clinical parameters	Score			Comments
	- 1	0	1	
Fever >101.3F(38.5C)	No/Unknown	Yes		
Lymphadenopathy		No/Unknown	Yes(+1)	>1 cm, at least 2 sites
Eosinophilia > 0.7 x10⁹ or ≥10% if leucopenia		No/Unknown	Yes(+2)	Score 2 points of ≥1.5 × 109
Atypical lymphocytes		No/Unknown	Yes	
Rash suggestive of DRESS	No(-1)	Unknown	Yes	Suggestive features: ≥2 facial edemas, purpura, infiltration, desquamation
Rash Extent ≥50% of BSA		No/Unknown	Yes	
Skin biopsy suggestive of DRESS	No	Yes/Unknown		
Organ involvement		No	Yes(+1)	1 point for each organ involvement, maximum score: 2
Disease duration ≥15 days	No/Unknown	yes		
Exclusion of other causes		No/Unknown	yes	1 point if 3 of the following tests are performed and are negative: HAV, HBV, HCV, mycoplasma, chlamydia, ANA, blood culture

**Figure 4:**
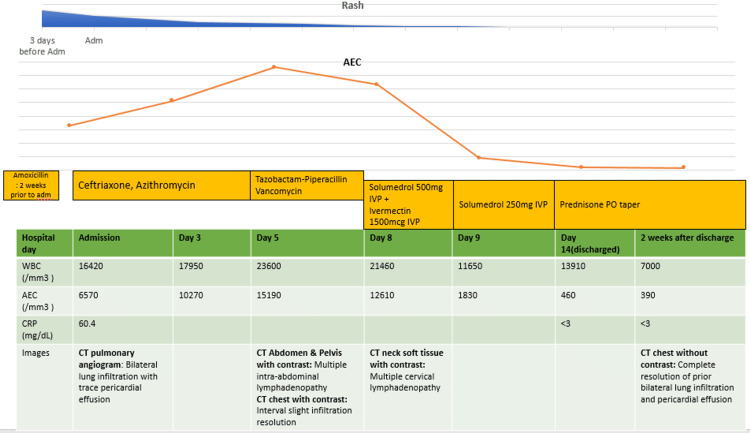
Clinical manifestations and hospital courses WBC: white blood cell; CRP: C-reactive protein; Adm: admission; IVP: intravenous push; PO: per oral

The decision to administer steroids was made after conducting an extensive workup to rule out other potential causes of HE. Referrals were made to the pulmonary and hematology-oncology services for further evaluation. After initiating steroid therapy, the patient's symptoms improved, and her AEC normalized within six days of starting treatment. Subsequently, the patient was discharged from the hospital on a steroid taper regimen and scheduled for follow-up appointments with multiple specialties. The above hospital courses are described in Figure 4.

During the follow-up period, the patient remained clinically stable, and a repeat CT scan of the chest showed complete resolution of bilateral lung infiltration (Figure 1C). The results of the workup were inconclusive, except for elevated levels of IgE (immunoglobulin E) and polyclonal gammopathy detected on serum protein electrophoresis (SPEP) (Table 3).

**Table 3:** Workup results for HE Significant for high IgE and polygammopathy on SPEP HE: hypereosinophilia; SPEP: serum protein electrophoresis

	Reference Value	Reference Units	Patient’s Value
Vitamin B12	232 - 1245	pg/mL	1014
Tryptase	≤ 8.2	ug/L	7.7
Trichinella Ab	Negative		Negative
Strongyloides Antibodies	Negative		Negative
Toxocari Cani Antibodies	Negative		Negative
Aspergillus Galactomannan Antigen	0.00 - 0.49	Index	0.08
Oval and Parasite Stool Screen (2 sets)			No Protozoa seen
Filaria IgG4 Antibody	<2.50	Index	2.53
Histoplasmosis Serology	<1:8		1:8
Sputum Culture			Normal Respiratory Flora
Blood Culture (4 sets)			No Growth Final
Coccidioides Antibodies	Negative		Negative
HIV AG/AB Screen	Non-Reactive		Non-Reactive
Hepatitis B Surface Antigen	Non-Reactive		Non-Reactive
Hepatitis B surface Antibody	Non-Reactive		Non-Reactive
Hepatitis B Core Antibody(total)	Non-Reactive		Reactive
Hepatitis B DNA	Not Detected		Not Detected
Hepatitis C Antibody	Non-Reactive		Non-Reactive
Quantiferon Tb	Negative		Indeterminate
IG (Immunoglobulin) E	≤ 100	KU/L	3922
IgG4 Subset	4 2 - 96	mg/dL	173
C3 Complement	81 - 157	mg/dL	127
C4 Complement	13 - 39	mg/dL	9
Anti dsDNA Ab	≤ 29	IU/mL	<12
ANA (Antinuclear Antibodies) Titer	<1:80		1:80 (speckled pattern)
Cytoplasmic Antineutrophil Cytoplasmic Antibodies (C-ANCA) AB	Negative		Negative
Perinuclear Antineutrophil Cytoplasmic Antibodies (P-ANCA) AB	1:20		1:80
ANCA (Antineutrophil Cytoplasmic Antibodies) Reflex Proteinase 3 Ab	≤ 20.0	Units	<5
Myeloperoxidase Ab	≤ 20.0	Units	<5
Cyclic Citrullinated Peptide Ab	≤ 19	Units	<8
Anti-Ribonuclear Protein	≤ 0.9	AI	0.4
Scleroderma Antibodies	≤ 0.9	AI	<0.2
Sjogren's SS-A AB (RO)	≤ 0.9	AI	<0.2
Sjogren's SS-B AB (LA)	≤ 0.9	AI	<0.2
Centromere Antibodies	≤ 0.9	AI	<0.2
Rheumatoid Factor	0 - 13	IU/mL	12
Fluorescence In Situ Hybridization (FISH) Analysis	Normal FISH - BCR/ABL1, MPD PANEL		
Chromosome Analysis	No evidence of clinically significant numerical or structural chromosome abnormalities		
Protein Electrophoresis, Serum	Polyclonal gammopathy. No monoclonal antibody detected.		
Flow Cytometry	No diagnostic abnormality on lymphocyte immunophenotypic findings. Increased proportion of eosinophils on the myeloid immunophenotypic findings.		

## Discussion

Drug reaction with eosinophilia and systemic symptoms (DRESS) syndrome is a delayed, infrequent, potentially life-threatening idiosyncratic drug reaction [1,2]. It has a reported mortality rate of 5% to 10%, and approximately 11.5% of patients experience long-term sequelae, with autoimmune thyroid diseases being the most common [3]. While aromatic anticonvulsants and allopurinol are the most frequent causative agents, there are also reports of antibiotic-induced DRESS syndrome, although they are less common. Penicillin-induced DRESS accounts for less than 10% of reported cases of antibiotic-induced DRESS syndrome [4]. Additionally, there have been reports suggesting that amoxicillin may play a role in triggering DRESS syndrome in patients who already exhibit signs of intolerance to sulfasalazine. However, the role of amoxicillin in the development of DRESS syndrome in individuals with no previous history of the condition remains unclear [4]. It is worth noting that cases of amoxicillin-induced DRESS syndrome as a standalone trigger have been rarely described in the literature. The patient with no known allergy to penicillin or sulfasalazine presented with an atypical rash, HE, likely organ involvement on CT chest, and generalized lymphadenopathy two to three weeks after taking amoxicillin. The admission medical records mentioned generalized maculopapular rashes on the extremities, back, and trunk, resembling the initial phase of a typical DRESS rash [5]. However, there was no photographic evidence to confirm this. The erythematous macules coalescing into patches that the patient had on admission day 6 might have represented the coalescing phase of the initial maculopapular rashes. Ultimately, one more point was scored by excluding other causes of HE with negative workup results, bringing the total score to 4 points, suggesting a probable DRESS syndrome.

The patient also presented with HE, according to its definition (Table 4) [6], even if the absolute eosinophil count (AEC) could not be measured at two-week intervals due to the acute worsening manifestations requiring early treatments. However, the patient did not meet the criteria for HES because a tissue biopsy was not performed in light of the improvement in lung infiltration with steroid treatment on the follow-up CT chest.

**Table 4:** Definitions of hypereosinophilia (HE) and hypereosinophilic syndrome (HES)

Term	Definition and criteria
Hypereosinophilia	≥1.5 eosinophils ×10⁹ /L peripheral blood on two examinations (interval ≥2 weeks)
Tissue Hypereosinophilia	One or more of the following applies: a) the percentage of eosinophils in bone marrow section exceeds 20% of all nucleated cells, and/or b) a pathologist is of the opinion that tissue infiltration by eosinophils is extensive and/or c) marked deposition of eosinophil granule proteins is found (in the absence or presence of tissue infiltration by eosinophils)
Hypereosinophilic syndrome	a) Criteria for blood HE fulfilled and: b) organ damage and/or dysfunction attributable to tissue HE and c) exclusion of other disorders or conditions as the major reason for organ damage

**Figure 5:**
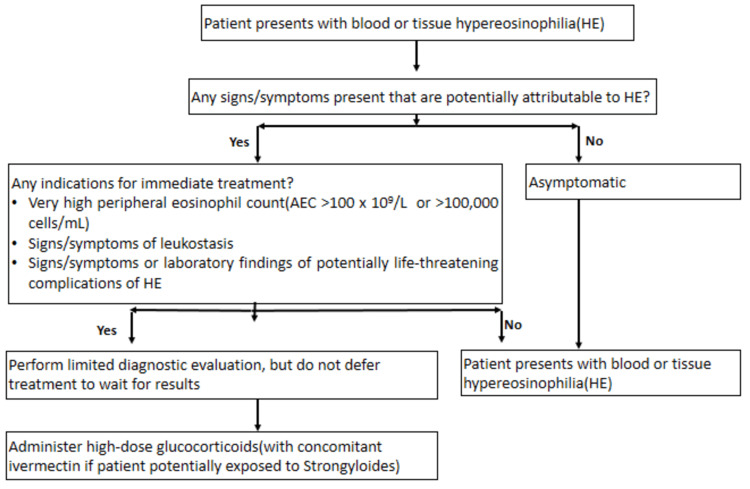
Algorithm for the immediate treatment of hypereosinophilia Potentially life-threatening complications of HE include: 1. Acute heart failure or evidence of likely developing eosinophil-mediated cardiac damage (based on either clinical findings or elevated troponins when clinical cardiac impairment is not yet clinically evident). 2. Thromboemboli, often of cardiac origin, which can present as diffuse watershed central nervous system (CNS) infarctions or focal CNS thromboemboli. 3. Pulmonary signs or symptoms, in concert with substantial eosinophil-associated disease on computed tomography (CT) imaging of the chest.

DRESS syndrome associated with significant HE requires exclusive diagnosis by comprehensive investigation of other causes of HE (Table 5) [6]. Immediate high-dose steroid treatment is typically initiated in cases where patients have life-threatening symptoms while ongoing workups are conducted to determine appropriate second-line treatment options based on the underlying cause (Figure 5) [7-9].

**Table 5:** Variant of hypereosinophilia (HE)

Variant of HE	Features
Secondary (reactive) HE	Underlying reactive condition or disease that explains HE: adverse drug reactions, infections (e.g., helminth infections), inflammatory diseases, and lymphoid neoplasms (often T-cell neoplasms producing eosinopoietic cytokines), no evidence for a clonal bone marrow disease that explains HE, and no signs or symptoms indicative of HES
Hereditary (familial) HE	Familial clustering, often evidence of a hereditary immunodeficiency (inborn errors of immunity with eosinophilia), no evidence of a reactive or neoplastic underlying disease, and no signs or symptoms indicative of HES
HE of unknown significance	No known underlying etiology of HE, no positive family history, no evidence of a reactive or neoplastic condition or disorder underlying HE, and no signs or symptoms indicative of HES
Clonal (neoplastic) HE	Underlying stem cell, myeloid, or eosinophil neoplasm inducing HE; no signs/symptoms indicative of HES

The extensive workup came back inconclusive except for high levels of IG (immunoglobulin) E and polyclonal gammopathy on serum protein electrophoresis (SPEP). IgE levels are typically elevated in allergic reactions like type 1 hypersensitivity, lymphoid-HES (LHES), EGPA (eosinophilic granulomatosis with polyangiitis, i.e., Churg-Strauss syndrome), and some immunodeficiencies associated with eosinophilia (e.g., hyper-IgE syndrome) [10]. The patient did not have a history of asthma or sinusitis suggestive of EGPA. ANA (antinuclear antibody) titer was borderline at 1:80 with elevation p-ANCA (antineutrophil cytoplasmic antibodies) titer of 1:80, but later both results normalized on the repeated test. Antibodies to proteinase-3 (PR3), rheumatoid factor, and other autoimmune disease serologies were negative. The patient did not have a constellation of symptoms and signs indicative of hyper-IgE syndrome such as coarse facial features. No diagnostic abnormalities on the lymphocyte immunophenotypic findings suggestive of LHES were noted on flow cytometry. In addition, the elevated IgG4 level was not high enough to meet the indication for a diagnostic evaluation of IgG4-RD (related diseases) [11]. Also, polyclonal gammopathies are most often due to infectious, inflammatory, or reactive processes, which could be attributed to the patient's acute conditions [12]. For other test results, FISH (fluorescence in situ hybridization) analysis did not show abnormal BCR/ABL1 or myeloproliferative disease (MPD) panel indicative of myeloid diseases. Parasite serologies and stool parasite screening were unremarkable. The patient had a negative QuantiFERON result one year ago, but it turned out to be ‘indeterminate’ this time. Nevertheless, given the improvement in symptoms and repeat CT chest findings, active tuberculosis was considered less likely.

Based on the comprehensive workup results, clinical correlations, and significant improvement with steroids, it is concluded that the patient likely had amoxicillin-induced DRESS syndrome presenting as reactive hypereosinophilia (HE). Close monitoring of the patient's condition is necessary, as a single negative test result cannot completely rule out other potential differential diagnoses. It is important to note that penicillins, including amoxicillin, can elicit hypersensitivity reactions of various types, ranging from type 1 to type 4 [13]. In this case, the patient exhibited DRESS syndrome, which is a type 4 reaction. The presence of a high level of IgE could suggest the possibility of an additional IgE-mediated (type 1) drug reaction. This could be attributed to the continuous exposure to another penicillin (piperacillin-tazobactam) and a cross-reacting drug (ceftriaxone) administered during the admission for suspected pneumonia. The clinical deterioration with skin irritation further supports this suspicion.

## Conclusions

Distinguishing DRESS syndrome presenting as reactive HE from other disorders, including other causes of reactive HE, different variants of HE, and HES, is crucial. A thorough diagnostic evaluation is necessary to determine the underlying etiology and guide appropriate treatment. Prompt empirical treatment with high doses of steroids is essential for patients with potentially life-threatening symptoms, even before the exact cause is determined. This immediate treatment can help mitigate the severe manifestations of DRESS syndrome. However, it's important to continue investigating other potential causes of HE to ensure appropriate management tailored to the specific underlying condition. Clinicians should maintain a high index of suspicion for DRESS syndrome and be aware that amoxicillin alone can induce DRESS, independent of the role of beta-lactamase in triggering the syndrome. This highlights the importance of considering amoxicillin as a potential culprit in cases of suspected DRESS syndrome. Long-term monitoring is necessary for the development of autoimmune sequelae, as patients with DRESS syndrome are at risk of developing such complications. Strict avoidance of the offending drug and any cross-reacting drugs is crucial to prevent further episodes of DRESS syndrome or exacerbation of symptoms. Education and patient awareness regarding drug allergies, avoidance of specific medications, and recognition of potential symptoms of DRESS syndrome are important aspects of long-term management.
